# Atrial Anti-Arrhythmic Effects of Heptanol in Langendorff-Perfused Mouse Hearts

**DOI:** 10.1371/journal.pone.0148858

**Published:** 2016-02-12

**Authors:** Gary Tse, Vivian Tse, Jie Ming Yeo, Bing Sun

**Affiliations:** 1 School of Biomedical Sciences, Li Ka Shing Faculty of Medicine, University of Hong Kong, Hong Kong S.A.R., China; 2 Department of Physiology, McGill University, Montreal, Canada; 3 Faculty of Medicine, Imperial College London, London, United Kingdom; 4 Department of Cardiology, Tongji University Affiliated Tongji Hospital, Shanghai, China; University of Minnesota, UNITED STATES

## Abstract

Acute effects of heptanol (0.1 to 2 mM) on atrial electrophysiology were explored in Langendorff-perfused mouse hearts. Left atrial bipolar electrogram or monophasic action potential recordings were obtained during right atrial stimulation. Regular pacing at 8 Hz elicited atrial activity in 11 out of 11 hearts without inducing atrial arrhythmias. Programmed electrical stimulation using a S1S2 protocol provoked atrial tachy-arrhythmias in 9 of 17 hearts. In the initially arrhythmic group, 2 mM heptanol exerted anti-arrhythmic effects (Fisher’s exact test, *P* < 0.05) and increased atrial effective refractory period (ERP) from 26.0 ± 1.9 to 57.1 ± 2.5 ms (ANOVA, *P* < 0.001) despite increasing activation latency from 18.7 ± 1.1 to 28.9 ± 2.1 ms (*P* < 0.001) and leaving action potential duration at 90% repolarization (APD_90_) unaltered (25.6 ± 1.2 vs. 27.2 ± 1.2 ms; *P* > 0.05), which led to increases in ERP/latency ratio from 1.4 ± 0.1 to 2.1 ± 0.2 and ERP/APD_90_ ratio from 1.0 ± 0.1 to 2.1 ± 0.2 (*P* < 0.001). In contrast, in the initially non-arrhythmic group, heptanol did not alter arrhythmogenicity, increased AERP from 47.3 ± 5.3 to 54.5 ± 3.1 ms (*P* < 0.05) and activation latency from 23.7 ± 2.2 to 31.3 ± 2.5 ms and did not alter APD_90_ (24.1 ± 1.2 vs. 25.0 ± 2.3 ms; *P* > 0.05), leaving both AERP/latency ratio (2.1 ± 0.3 vs. 1.9 ± 0.2; *P* > 0.05) and ERP/APD_90_ ratio (2.0 ± 0.2 vs. 2.1 ± 0.1; *P* > 0.05) unaltered. Lower heptanol concentrations (0.1, 0.5 and 1 mM) did not alter arrhythmogenicity or the above parameters. The present findings contrast with known ventricular pro-arrhythmic effects of heptanol associated with decreased ERP/latency ratio, despite increased ERP/APD ratio observed in both the atria and ventricles.

## Introduction

The accurately timed sequence of contractile activity in different cardiac regions depends on the orderly generation and the subsequent propagation of an action potential (AP). Conduction velocity (CV) of the AP depends on sodium channel activation followed by gap junction conduction through successive myocardial regions [1–3]. Decreases in CV, which is proportional to activation latency, have been associated with increased risk of arrhythmias [4, 5]. Cardiac arrhythmias have been explored in murine models, which permit alterations in the expression or function of ion channels using genetic or pharmacological methods [6, 7]. The resulting electrophysiological abnormalities can then be characterized using both *in vivo* and *ex vivo* techniques [8, 9].

Recently, acute, reversible ventricular arrhythmias due to CV slowing were observed using the gap junction and sodium channel inhibitor heptanol in wild-type mouse hearts [10]. The present study goes on to examine its atrial effects under similar experimental conditions, and demonstrate contrasting electrophysiological properties under both control conditions and in the presence of heptanol. Heptanol at 2 mM exerted anti-arrhythmic effects, attributable to its differing actions on activation latency, ERP and APD and their consequent scaling of ERP/latency and ERP/APD ratios, thereby implicating these parameters as critical in the determination of arrhythmogenicity.

## Materials and Methods

All procedures described in this study complied with the UK Animals (Scientific Procedures) Act 1986. This study was approved by the Animal Welfare and Ethical Review Body at the University of Cambridge. All procedures described in this study complied with the UK Animals (Scientific Procedures) Act 1986. Both male and female wild-type mice (129 genetic background), between 5 and 7 months of age, were housed in plastic cages of an animal house facility at room temperature (21 ± 1°C) and subject to a 12:12 h light / dark cycle. The mice had access to sterile rodent chow and drinking water at all times. Krebs-Henseleit solution used in the experiments (mM: NaCl 119, NaHCO_3_ 25, KCl 4, KH_2_PO_4_ 1.2, MgCl_2_ 1, CaCl_2_ 1.8, glucose 10 and sodium pyruvate 2, pH 7.4) was bubbled with 95% O_2_−5% CO_2_ [11]. Heptanol (Sigma, Dorset, UK; density: 0.82 g ml^–1^), which is soluble in aqueous solutions up to 9 mM (The Merck Index, New Jersey, USA), was diluted using Krebs-Henseleit solution to produce final concentrations between 0.1 and 2 mM.

The Langendorff perfusion technique is an established method that has been adapted for study of cardiac electrophysiology in mice [12]. Mice were killed by cervical dislocation (Schedule 1 of the UK Animals (Scientific Procedures) Act 1986). This permitted isolation of their hearts, which were then submerged in ice-cold bicarbonate-buffered, Krebs-Henseleit solution. The surrounding lung tissue was removed, with the aorta cannulated using a tailor-made 21-gauge cannula prefilled with ice-cold buffer, secured using a micro-aneurysm clip (Harvard Apparatus, Kent, UK) and attached to the perfusion apparatus. The aorta was perfused with Krebs-Henseleit solution at a rate of 2 to 2.5 ml/min using a peristaltic pump (Watson–Marlow Bredel pumps model 505S, Cornwall, UK), passing successively through 200 μm and 5 μm filters and warmed to 37°C by water jacket and circulator. Hearts that regained their pink colour and began to contract spontaneously were studied further (approximately 90%). The remaining 10% were discarded. To minimize residual effects of endogenous release of catecholamines, the hearts were perfused with Krebs–Henseleit solution for a further 20 minutes before experimentation. The time taken for the perfusing solution to reach the heart from the buffer reservoir was determined from a series of control experiments, which involved the addition of a coloured solution to a colourless Krebs–Henseleit solution and measurement of the time taken for the discarded solution to gain coloration. This procedure was repeated twice and the mean duration was calculated. Hearts were studied for similar durations of heptanol exposure, which were 360 ± 51 s, 314 ± 32 s, 312 ± 54 s and 380 ± 56 s for 0.1 mM, 0.5 mM, 1 mM and 2 mM heptanol, respectively (*n* = 11). Because of the known time-dependent effects of heptanol in the ventricles [10], the electrophysiological parameters were consistently analyzed after a standardized time point which was taken to be 120 seconds after its introduction.

Electrical stimulation of the right atrial epicardium was achieved using paired platinum electrodes (1 mm inter-pole distance). Regular pacing was set at 8 Hz (i.e. basic cycle length, (BCL), of 125 ms), using 2 ms duration, square-wave stimuli applied at three times the excitation threshold (Grass S48 Stimulator, Grass-Telefactor, Berkshire, UK), allowing direct comparison with previous mouse studies of atrial and ventricular arrhythmogenesis [10, 13, 14].

Programmed electrical stimulation (PES) imposed drive trains of eight paced S1 stimuli delivered at a 125 ms BCL, followed by premature S2 extra-stimuli every ninth stimulus. S1S2 intervals first equalled the pacing interval and were then successively reduced by 1 ms with each stimulus cycle, until the atrial effective refractory period (AERP) was reached or arrhythmic activity was provoked. PES was applied twice before application of the test agent. It was then applied at standardized time points of 120 seconds after its application, because of its known time-dependent effects on activation latency and ERP in the ventricles [10]. This duration was sufficient for the actions of heptanol to take place, as previously shown. PES was applied 15 minutes after its removal from the perfusing solution to allow reversibility of heptanol to be studied. In between PES procedures hearts were regularly paced at 8 Hz.

Bipolar electrograms (BEGs) were recorded from the left atrial epicardium using a paired (1 mm inter-pole spacing) platinum electrode. Monophasic action potentials (MAPs) were also recorded from the left ventricular epicardium using a MAP electrode (Linton Instruments, Kent, UK). Such simultaneous recordings permitted atrial activity to be distinguished from ventricular fari-field activity at the atrial recording electrode. The BEG signals were pre-amplified (NL100AK head stage), amplified (NL 104A amplifier) and filtered (band-pass between 30 Hz and 1 kHz) using a NL125/6 filter (Neurolog, Hertfordshire, UK) and then digitized using an analogue-to-digital converter (1401plus MKII, Cambridge Electronic Design, Cambridgeshire, UK) at 5 kHz. For atrial MAP recordings, the atrio-ventricular nodes of the Langendorff perfused hearts were first mechanically ablated as previously described [13, 14]. This eliminated ventricular far-field activity at the recording electrode. MAPs were recorded from the left atrial epicardium during regular 8 Hz stimulation to exclude rate-dependent differences in action potential durations (APDs). All MAPs were pre-amplified, amplified and band-pass filtered between 0.5 Hz and 1 kHz and then digitized at 5 kHz. The waveforms were then analysed using Spike2 software (Cambridge Electronic Design, Cambridgeshire, UK). MAP waveforms not matching the criteria for MAP signals of a stable baseline, fast upstroke without inflection or negative spike and rapid first phase of repolarization were rejected [15]. 0% repolarization was measured at the peak of the MAP and 100% repolarization was measured at the point of return of the membrane potential to baseline [15–17].

The following atrial electrophysiological parameters were derived from the experimental recordings:

(1) activation latency, defined as the time difference between the stimulus artefact and the first deflection of the BEG; (2) AERP, defined as the longest S1S2 interval at which the S2 extrastimulus failed to initiate an atrial signal during PES; (3) APD_x_, defined as the time taken for the recorded voltage to decline from the peak of the MAP to x% repolarization; (4) AERP/latency ratio and (6) AERP/APD_90_ ratio.

All experimental values obtained are given as mean ± standard error of the mean (SEM). Comparisons between different experimental groups were carried out by one-way analysis of variance (ANOVA) followed by Tukey’s Honestly Significant Difference test, and Student’s *t*-test as appropriate. Categorical data were compared with Fisher’s exact test (two-tailed). *P* < 0.05 was considered statistically significant in all cases. *P* < 0.05, *P* < 0.01 and *P* < 0.001 were denoted by *, ** and ***, respectively.

## Results

The present experiments investigated the effects of the gap junction and sodium channel inhibitor heptanol (0.1, 0.5, 1 or 2 mM) on atrial arrhythmic and electrophysiological properties in Langendorff-perfused mouse hearts, complementing a previous report describing its ventricular pro-arrhythmic effects [10].

### Heptanol (2 mM) slows conduction without inducing atrial arrhythmic activity during regular pacing

**Fig 1:**
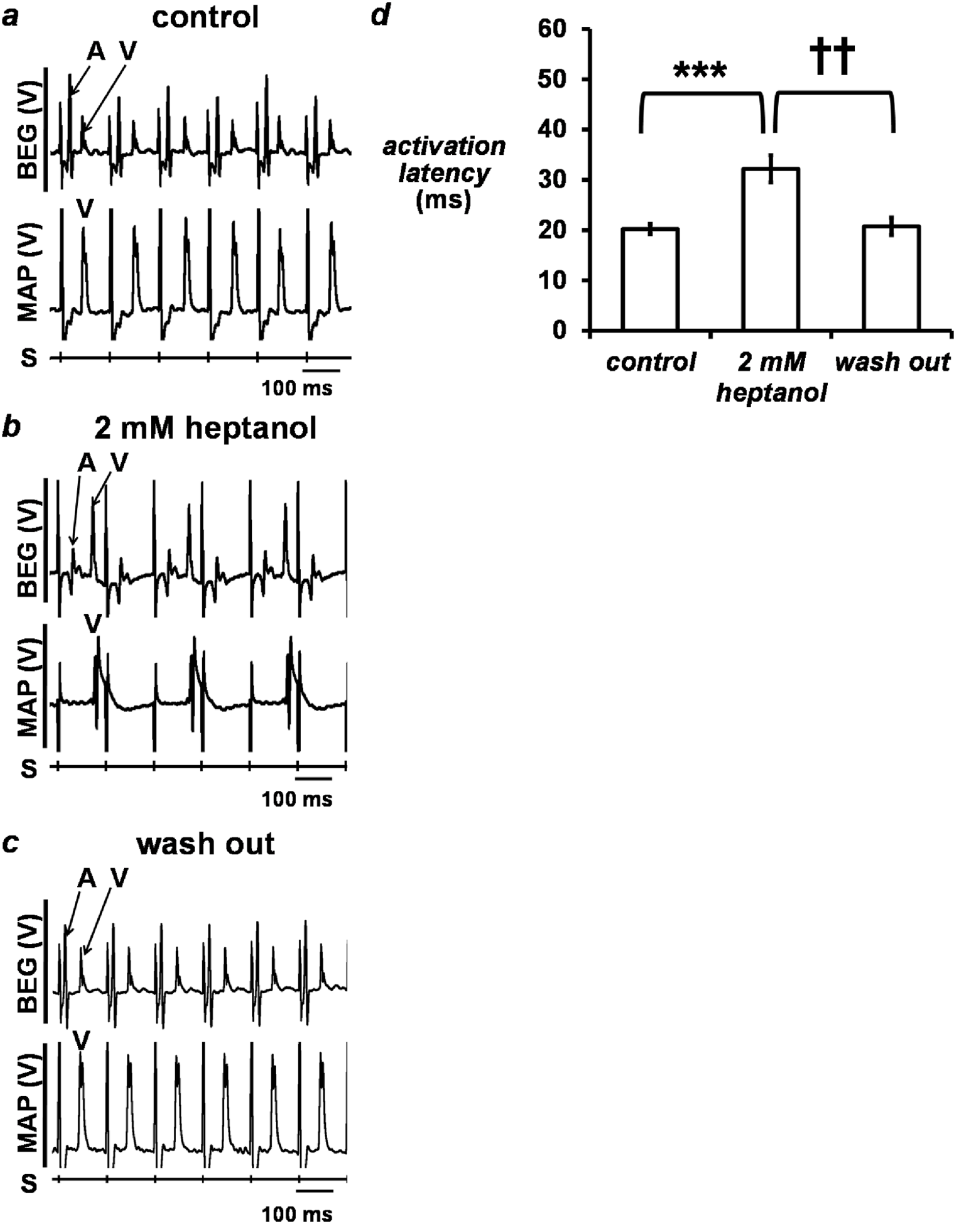
Simultaneous left atrial bipolar electrogram (BEG) (*top trace*) and left ventricular monophasic action potential (MAP) (*bottom trace*) recordings obtained before (a, control) and after the introduction (b) of 2 mM heptanol, and 15 minutes after its removal from the perfusing solution (c, wash out) during regular 8 Hz pacing. Atrial and ventricular deflections were labelled “A” and “V”, respectively. Activation latency (d) and CV (e) obtained before and after introduction of 2 mM heptanol, and 15 minutes after its removal (*n* = 11 in all cases). Activation latency was significantly increased (asterisks, ANOVA, *P* < 0.001) and therefore CV was significantly decreased (asterisks, *P* < 0.01) by 2 mM heptanol. The effects of heptanol were reversible as both activation latency and CV recovered to their control values after its removal from the perfusing solution (daggers, *P* > 0.05).

The initial experiments were conducted during regular pacing at 8 Hz, close to the mouse *in vivo* heart rate [18]. The hearts were exposed to Krebs-Henseleit solution (KHS) for 20 min, treated with 2 mM heptanol/KHS for at least 5 min, then returned to KHS alone for 15 min. Simultaneous bipolar electrogram (BEG) and monophasic action potential (MAP) recordings were obtained from the left atrial and the left ventricular epicardium, respectively, during this procedure (Fig 1a to 1c). Signals from atrial events were identified by deflections present in the BEG but absent from the MAP recordings, whereas those from ventricular events were identified by deflections present in both the BEG and MAP recordings (labelled “A” and “V”, respectively). It was therefore possible to distinguish atrial activity from ventricular far-field activity at the atrial recording electrode.

Regular atrial activity was observed before introduction of the test agent. Heptanol (2 mM) did not elicit any arrhythmic activity in the atria, despite gradually increasing activation latency from 20.2 ± 1.0 ms to 32.2 ± 2.7 ms (Fig 1d; asterisks, ANOVA, *P* < 0.001) over 120 seconds after its introduction (*n* = 11). Withdrawal of heptanol restored activation latency to 20.7 ± 1.3 ms, which were indistinguishable from control values (*P* > 0.05).

### A subset of control hearts shows inducible atrial tachy-arrhythmias which are inhibited by heptanol (2 mM)

**Fig 2:**
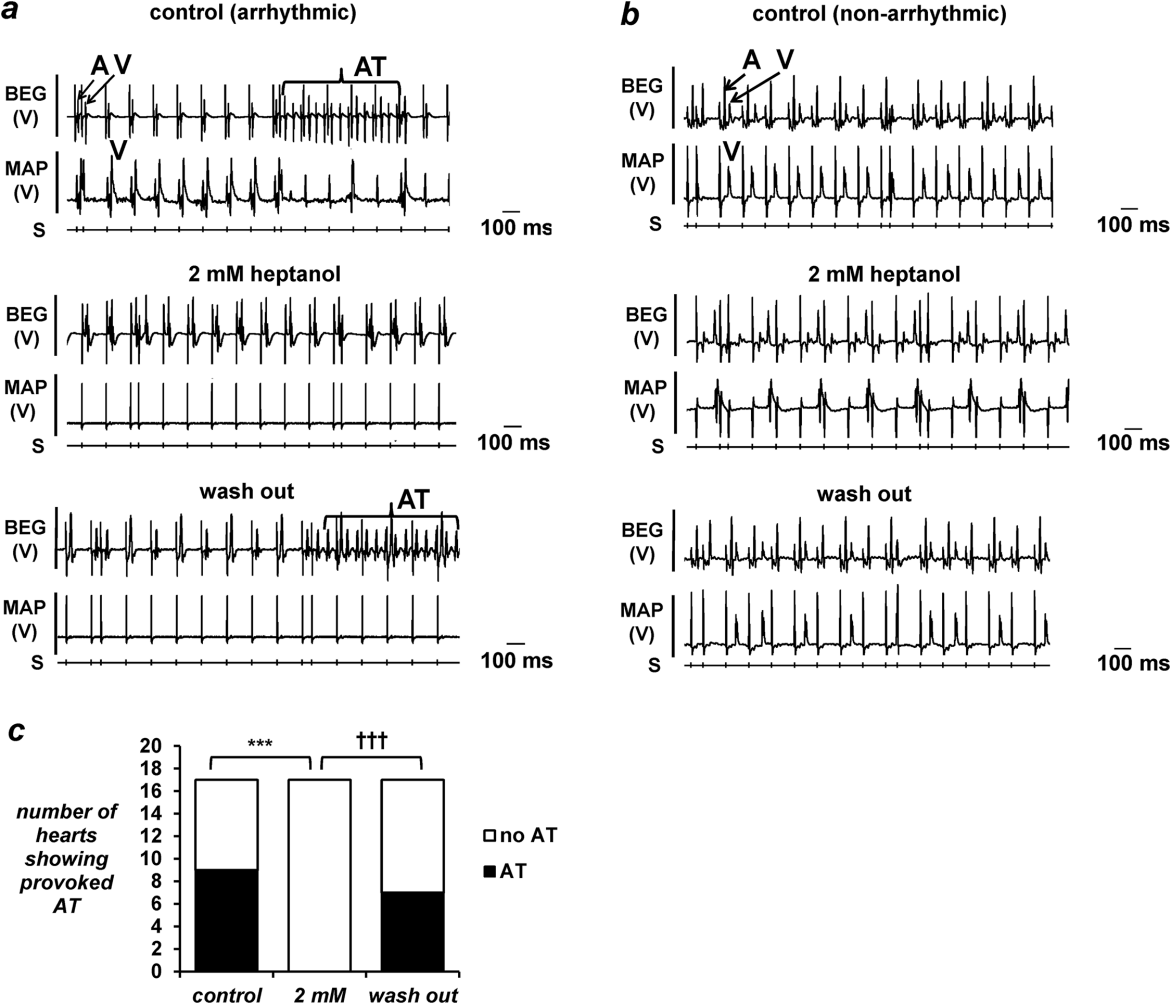
Simultaneous atrial BEG and ventricular MAP recordings obtained from a representative heart that showed provoked atrial tachy-arrhythmias during programmed electrical stimulation (PES) (a) before introduction of heptanol (*top panel*). Atrial and ventricular deflections were labelled “A” and “V” respectively, and the train of atrial tachy-arrhythmias was labelled “AT”. Recordings obtained after introduction of 2 mM heptanol showed that AT could not be provoked during PES (*middle panel*). Recordings obtained 15 minutes after removal of 2 mM heptanol from the perfusing solution showing the return of atrial tachy-arrhythmias (*bottom panel*). Simultaneous atrial BEG and ventricular MAP recordings obtained from a representative heart that did not show AT during PES (b) before introduction of 2 mM heptanol (*top panel*). Recordings obtained seconds after its introduction (*middle panel*) and 15 minutes after its removal (*bottom panel*) showed no change in atrial arrhythmogenicity. Incidence of atrial arrhythmias (c): heptanol (2 mM) exerted significant anti-arrhythmic effects (asterisks, Fisher’s exact test, *P* < 0.001) that were reversed upon its removal (daggers, comparison between 2 mM and washout, *P* < 0.001).

**Fig 3:**
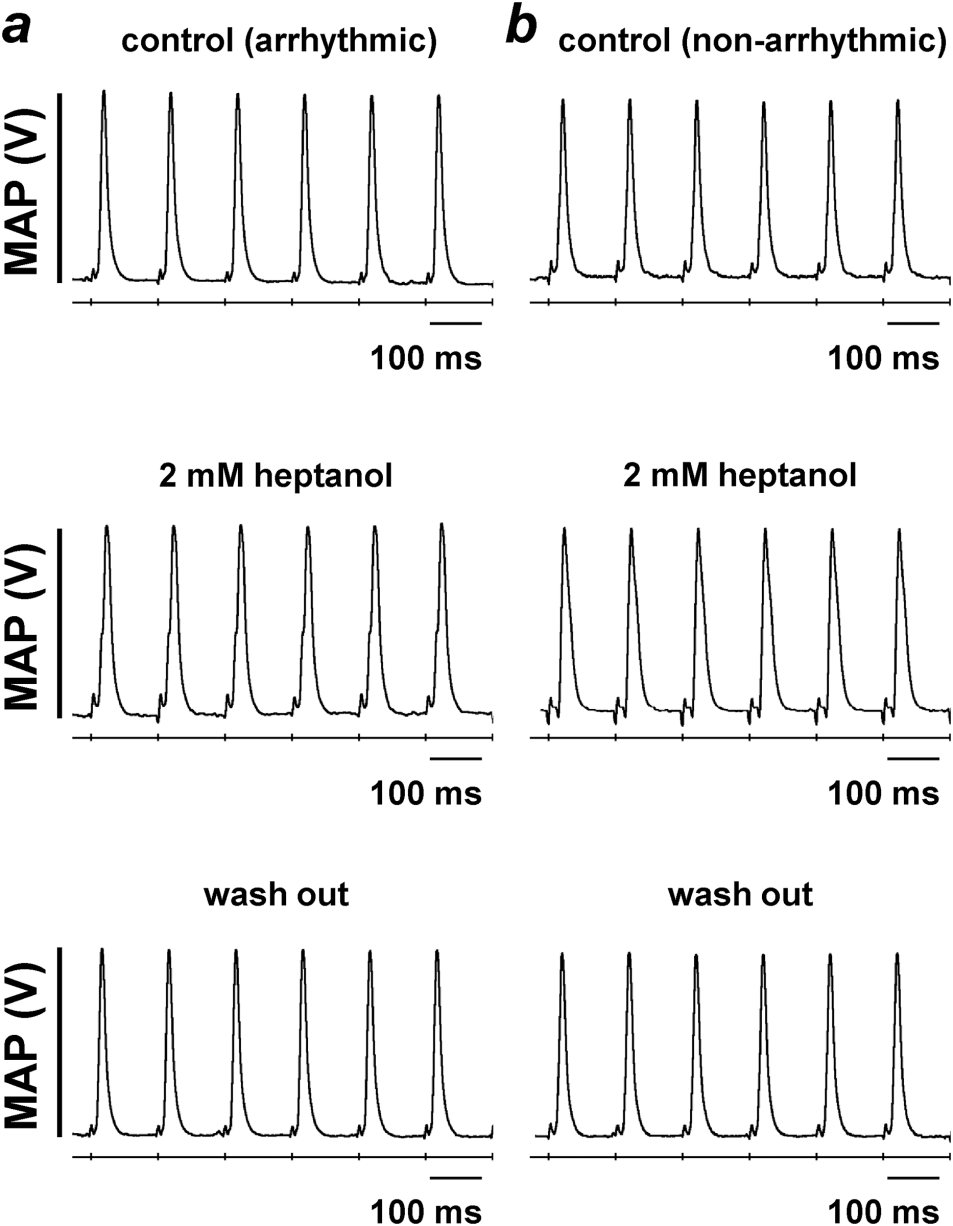
Monophasic action potential (MAP) recordings obtained from the arrhythmic (a) and non-arrhythmic hearts (b) during regular 8 Hz pacing before (*top panel*) and after introduction of 2 mM heptanol (*middle panel*) and after its removal from the perfusing solution (*bottom panel*).

The subsequent experiments examined the incidences of inducible arrhythmias using PES procedures under the same pharmacological conditions as above. This identified a subset of the hearts showing episodes of atrial tachy-arrhythmias (Fig 2a, *top panel*). These arrhythmic phenomena were inhibited by heptanol (2 mM) (Fig 2a, *middle panel*), but subsequently reappeared after its removal from the perfusing solution (Fig 2a, *bottom panel*). The remaining hearts did not show evidence of atrial arrhythmias under control conditions (Fig 2b, *top panel*), and remained non-arrhythmic both after application (Fig 2b, *middle panel*) and withdrawal of heptanol (2 mM) from the perfusing solution (Fig 2b, *bottom panel*). In both the arrhythmic and non-arrhythmic groups, heptanol (2 mM) produced second degree atrioventricular block (AVB), which rapidly progressed to third degree AVB. The incidences of atrial arrhythmias are summarized in Fig 2c: 9 out of 17 hearts were arrhythmic under control conditions and all 9 were rendered non-arrhythmic by heptanol (2 mM) (Fisher’s exact test, *P* < 0.001). Atrial tachy-arrhythmias reappeared in 7 of these hearts after its withdrawal from the perfusing solution (Fisher’s exact test, *P* < 0.001). Finally, atrial MAP recordings were obtained during 8 Hz pacing (Fig 3), allowing action potential durations (APDs) to be determined.

### Heptanol increases activation latency and ERP without altering APD in both the arrhythmic and non-arrhythmic groups

**Fig 4:**
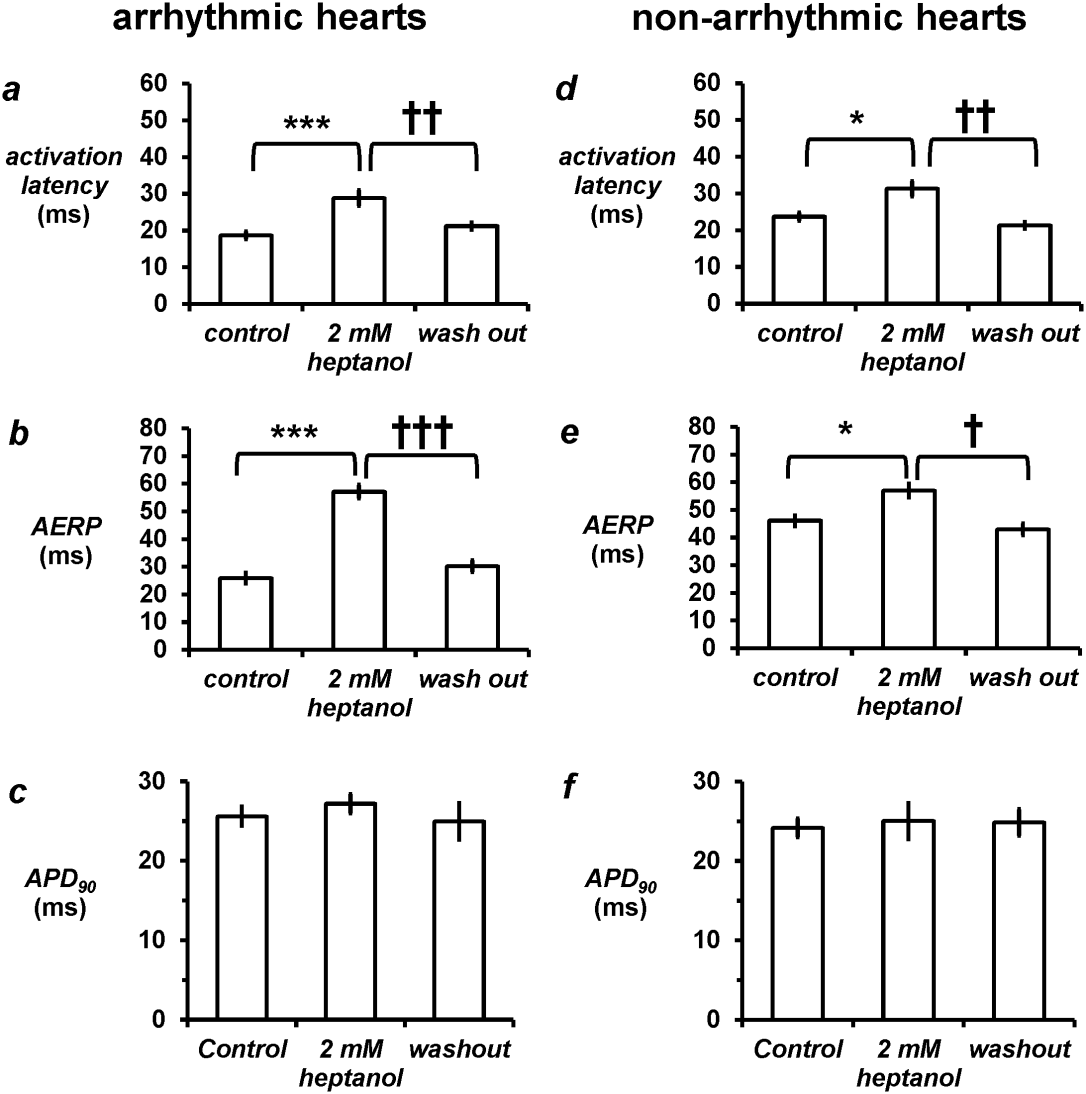
In the arrhythmic group, 2 mM heptanol increased activation latency (a, asterisks, ANOVA, *P* < 0.001, *n* = 9) and therefore decreased the corresponding CV (b; *P* < 0.01; *n* = 9) and increased atrial effective refractory period (AERP) (c, *P* < 0.001; *n* = 9) without altering APD_90_ (d, *P* > 0.05, *n* = 5). In the non-arrhythmic group, 2 mM heptanol also increased activation latency (e, *P* < 0.05, *n* = 8), decreased CV (f, *P* < 0.05, *n* = 8) and increased AERP (g, *P* < 0.05, *n* = 8) without altering APD_90_ (h, *P* > 0.05, *n* = 5). All values recovered to control values after removal of heptanol from the perfusing solution (*P* > 0.05).

The arrhythmic group had a mean activation latency of 18.7 ± 1.1 ms, AERP of 26.0 ± 1.9 ms and APD_90_ of 25.6 ± 1.2 ms (Fig 4a, 4b and 4c). Heptanol increased activation latency to 28.9 ± 2.1 ms (*asterisks*, ANOVA, *P* < 0.001), and AERP to 57.1 ± 2.5 ms (*asterisks*, *P* < 0.001) without altering APD_90_ (27.2 ± 1.2 ms; *P* > 0.05). Compared to the arrhythmic group, the non-arrhythmic group showed longer activation latency (Fig 4d; 23.7 ± 2.2 ms; *P* < 0.05) but longer AERP (Fig 4e; 47.3 ± 5.3 ms; *P* < 0.001) and similar APD_90_ (Fig 4f; 24.1 ± 1.2 ms). In this group, heptanol (2 mM) increased activation latency to 31.3 ± 2.5 ms (asterisk, *P* < 0.05), and increased AERP to 54.5 ± 3.1 ms (*asterisk*, *P* < 0.05) without altering APD_90_ (25.0 ± 2.3 ms; *P* > 0.05). Activation latency, AERP and APD_90_ values in both the arrhythmic and non-arrhythmic groups recovered to their baseline values after withdrawal of heptanol (*P* > 0.05).

### Heptanol increases AERP/latency and AERP/APD ratios in the arrhythmic group but not in the non-arrhythmic group

**Fig 5:**
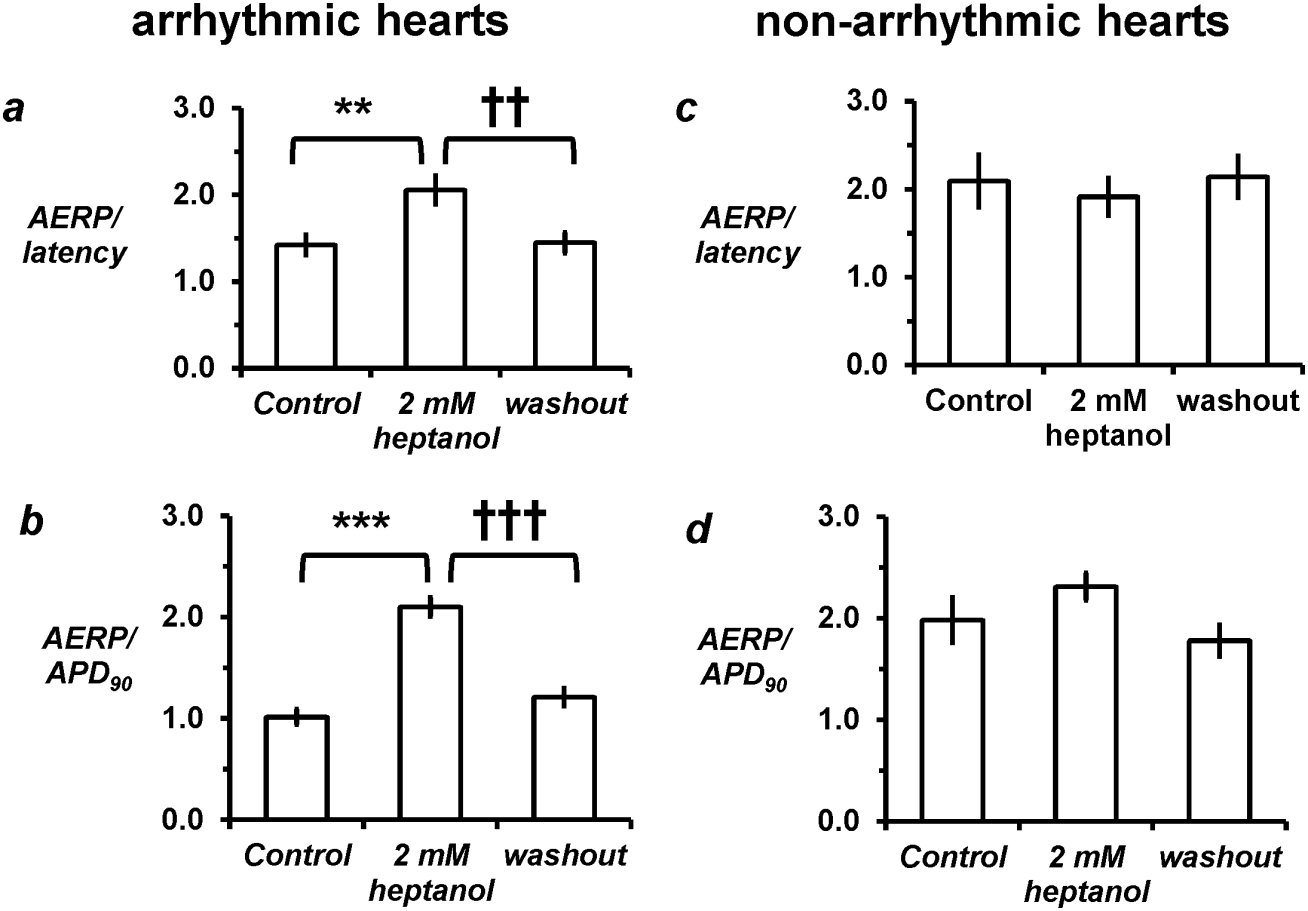
Derived parameters. Heptanol (2 mM) increased excitation wavelength (a, asterisks, ANOVA, *P* < 0.01) and AERP/APD_90_ ratio (b, *P* < 0.001) in the arrhythmic group, but did not alter either parameter in the non-arrhythmic group (c and d, *P* > 0.05). All values recovered to control values after removal of heptanol from the perfusing solution (*P* > 0.05).

In the arrhythmic group, heptanol (2 mM) increased AERP/latency ratio from 1.4 ± 0.1 to 2.1 ± 0.2 (*asterisks*, *P* < 0.01) and increased AERP/APD_90_ ratio from 1.0 ± 0.1 to 2.1 ± 0.2 (*asterisks*, *P* < 0.001) (Fig 5a and 5b). Baseline characteristics were also different for these derived parameters in the non-arrhythmic group, in that it showed longer AERP/latency ratio (Fig 5c; 2.1 ± 0.3; *P* < 0.05) and higher AERP/APD ratio (Fig 5d; 2.0 ± 0.2; *P* > 0.05) compared to the initially arrhythmic group. Heptanol did not significantly alter either parameter in this group (*P* > 0.05). In both the arrhythmic and non-arrhythmic groups, AERP/latency and ERP/APD ratio recovered to their baseline values after withdrawal of heptanol.

In a separate set of experiments, the concentration-dependence of heptanol were explored. Lower heptanol concentrations did not show any anti-arrhythmic effects, nor were any changes in CV, ERP, APD, AERP/latency and AERP/APD ratios observed (Table 1).

**Table 1:** Concentration-dependence of incidence of spontaneous and provoked atrial arrhythmia, APD_x_ values, activation latency, AERP, AERP/latency and AERP/APD_90_ ratios.

Parameter	heptanol concentration (mM)									*n* (all concentrations)
	control	0.1	*P*	0.5	*P*	1	*P*	2	*P*	
spontaneous arrhythmia	0 out of 11	0 out of 11	N.S.	0 out of 11	N.S.	0 out of 11	N.S.	0 out of 11	N.S.	11
inducible arrhythmia	5 out of 10	2 out of 10	N.S.	4 out of 10	N.S.	4 out of 10	N.S.	0 out of 10	< 0.05	10
APD_90_	25.2 ± 1.0	24.3 ± 1.6	N.S.	24.6 ± 1.3	N.S.	25.6 ± 1.1	N.S.	25.8 ± 1.8	N.S.	7
APD_70_	13.9 ± 0.5	12.6 ± 1.8	N.S.	13.8 ± 1.3	N.S.	15.7 ± 1.2	N.S.	14.0 ± 1.3	N.S.	7
APD_50_	8.2 ± 0.9	8.3 ± 1.8	N.S.	9.2 ± 1.3	N.S.	9.9 ± 1.3	N.S.	8.6 ± 1.2	N.S.	7
APD_30_	4.3 ± 0.5	5.2 ± 1.0	N.S.	5.8 ± 1.0	N.S.	6.6 ± 1.1	N.S.	5.6 ± 1.0	N.S.	7
activation latency	21.9 ± 1.3	23.0 ± 3.1	N.S.	21.9 ± 2.8	N.S.	23.9 ± 3.1	N.S.	29.5 ± 1.8	< 0.01	10
AERP	34.4 ± 2.8	32.9 ± 4.7	N.S.	39.6 ± 5.8	N.S.	41.1 ± 6.1	N.S.	60.0 ± 1.9	< 0.001	10
AERP/latency ratio	1.6 ± 0.1	1.5 ± 0.1	N.S.	1.8 ± 0.2	N.S.	1.8 ± 0.2	N.S.	2.1 ± 0.2	< 0.05	10
AERP/APD ratio	1.3 ± 0.1	1.4 ± 0.2	N.S.	1.6 ± 0.2	N.S.	1.6 ± 0.2	N.S.	2.3 ± 0.1	< 0.001	7

Taken together, the above findings demonstrate that heptanol exerted atrial anti-arrhythmic actions, in contrast with its known ventricular pro-arrhythmic effects. In both the atrial and ventricles, increased activation latency, increased ERPs and unchanged APDs were found. These differences can nevertheless be explained by increased atrial but reduced ventricular ERP/latency ratio, despite increased ERP/APD ratios in both.

## Discussion

Cardiac excitation depends on an orderly activation and recovery sequences of action potentials (APs) through successive regions of the working myocardium [19]. Activation involves generation of the AP upstroke with a resulting activation latency that is inversely proportional to the conduction velocity (CV) [20]. Recovery involves both repolarisation and resumption of excitability, represented by action potential duration (APD) and effective refractory period (ERP), respectively. Disruption in the relationships between these electrophysiological parameters, for example due to reduced CVs, prolonged or shortened APDs or shortened ERPs, or combinations of such changes, can lead to both atrial and ventricular arrhythmias [21, 22]. This may be explained by a reduction in excitation wavelength given by CV x ERP, which would predispose the hearts to circus-type or spiral wave re-entry [23, 24]. In clinical electrophysiological studies, activation latency is often measured without determining the corresponding CVs (Katsiva *et al*., 1998). As such, excitation wavelength can be approximated using the ERP/latency ratio [25]. Alternatively, an APD/ERP ratio > 1 (i.e. a positive critical interval for re-excitation given by APD—ERP) may increase the propensity of re-excitation before full AP repolarization [26] via a phase 2 re-entrant mechanism [27] or one involving prolonged repolarization-dependent re-excitation [28].

Pharmacological methods have been used to study the mechanisms of arrhythmogenesis because these have the advantage of producing acute effects that can be reversed after drug withdrawal. For example, the lipophilic agent heptanol was used to explore the arrhythmogenic consequences of slowed AP conduction in mouse [10, 29], rat [30], rabbit [31, 32] and canine heart preparations [33]. It reduces gap junction conductance at ≤ 1 mM by decreasing the fluidity of cholesterol-rich domains in the plasma membrane [34]. It is also known to reduce sodium channel conductance ≥ 2 mM by producing both a depolarizing shift in the voltage dependence of activation and a hyperpolarizing shift in the voltage dependence of inactivation [35]. The inhibitory effects of heptanol on gap junctions and sodium channel provide an explanation for its action in reducing CV [10, 32]. Furthermore, prolongation of ERP can be explained by heptanol causing slower recovery of sodium channels from inactivation.

The present experiments proceeded to determine whether atrial properties differ from the ventricular findings both before and after heptanol treatment. There was no evidence of arrhythmias observed during regular pacing in the atria, as was the case in the ventricles in untreated hearts [10]. However, PES procedures provoked atrial tachy-arrhythmias in a subset of untreated hearts, consistent with previous observations of arrhythmia inducibility in the mouse atria [13, 14]. The arrhythmic group showed shorter activation latencies, shorter ERPs but statistically indistinguishable APDs when compared with the non-arrhythmic group. Therefore, lower ERP/latency and ERP/APD ratios were observed for the arrhythmic group, in keeping with the increased arrhythmogenicity.

It is interesting that under control conditions, there appears to be two distinct population of hearts, where one group showed an arrhythmic phenotype, whereas the other did not. Previous experiments in mouse hearts have demonstrated almost every heart showed evidence of atrial arrhythmias [13, 14]. However, the experiments described in these studies did not mention any exclusion criteria, implying that all of the hearts isolated were used for subsequent experimentation. It could very well be the case that some hearts in these experiments had endured tissue ischaemia during the isolation procedures, making the preparations more prone to arrhythmogenesis. In the present study, only the hearts that showed normal pink colouration and spontaneous ventricular activity were studied further, excluding the hearts that showed signs of myocardial damage during isolation that could have increased the baseline arrhythmogenicity under control conditions. In our previous studies, none of the hearts studied showed evidence of ventricular arrhythmias whether during regular pacing or PES [36]. The fact that atrial arrhythmias were observed in some hearts implied that the atria may be intrinsically more prone to arrhythmogenesis than the ventricles [10].

In contrast to its previously reported pro-arrhythmic ventricular effects, heptanol (2 mM) abolished these atrial arrhythmic phenomena, despite similarly increasing activation latencies and prolonging ERPs and leaving APDs unaltered in both cases. In the atria, it increased ERP/latency and ERP/APD ratios in the arrhythmic group to values that approximate those of the baseline non-arrhythmic group. Neither parameter was altered in the non-arrhythmic group with heptanol. Thus, heptanol rendered the arrhythmic hearts non-arrhythmic where their electrical properties shifted towards those of the non-arrhythmic group. Larger ERP/latency ratio would be expected to reduce circus-type or spiral wave entry, whereas increased ERP/APD ratio [37] would prevent phase 2 re-entry [38]. In the ventricles, pro-arrhythmic effects of heptanol were similarly associated with prolonged ERPs, increased activation latencies and unaltered APDs, with a resultant decrease in ERP/latency ratios despite increasing ERP/APD ratios. Moreover, the critical interval (CI) given by APD_90_ –ERP represents absolute time differences, rather than relative differences given by ERP/APD ratio. The CI represents the time period over which re-excitation is theoretically possible. Under control conditions, the CI was slightly negative in the arrhythmic atria and much more negative in the non-arrhythmic atria, and was also negative in the ventricles. It is therefore a poor predictor of arrhythmogenicity, because a negative interval should not be compatible with the occurrence of arrhythmias. Thus, consideration of these parameters in both atria and ventricles indicate that ERP/latency ratio appears to be the central determinant of arrhythmogenesis.

It is interesting to note that heptanol at all concentrations studied prolonged the interval between the start of atrial and the start of ventricular deflection. This would suggest heptanol exerting atrioventricular block. At the highest concentration studied of 2 mM, this block became a second degree heart block with 2:1 conduction, which rapidly progressed to third degree heart block within 90 seconds. The AV block produced by heptanol is consistent with its inhibitory effects on sodium channel and gap junction function, and the roles of these channels in normal AV conduction [39, 40].

### Limitations of this study

A major limitation study is that this study provides a phenomenological description of atrial and ventricular differences but cannot provide the evidence to explain these observations. Nevertheless, there are several potential explanations for the differences between atrial and ventricular electrophysiology. This may partly be attributed to heterogeneity in sodium channels in these tissues. In atrial myocytes, *I*_Na_ density is higher, activation and inactivation voltages are more negative, time constants for activation and inactivation are twice as rapid, and recovery from inactivation is slower when compared to ventricular myocytes [41]. The different kinetics of the sodium channel observed, in turn, could be explained by distinct α-subunit isoforms and further modulation by β-subunits [42–44]. Furthermore, differences in gap junctions mediating intercellular coupling are observed in these chambers. Thus, the Cx43 isoform is expressed in both atrial and ventricular tissue [45], whereas Cx40 is only found in the atria and His-Purkinje system [46] and not in the ventricles [47]. These different isoforms in atrial and ventricular tissue are responsible for distinct kinetics of the gap junction channels [48]. Together, the above properties could explain the difference in atrial and ventricular electrophysiology. Heptanol also affects atrial and ventricular tissue differently, exerting opposite effects on arrhythmogenicity. However, it is not known whether the kinetics of atrial and ventricular isoforms of gap junctions are affected differently by this agent. Secondly, CV were not determined in this study, as this would require measuring the distance between the stimulating and recording electrodes, which could be crudely measured using a ruler. A better method to record use a multi-electrode array, which would allow calculation of CVs by comparing activation times of adjacent sites in the array [49–51].

Finally, there does not appear to be a ‘cut-off’ ERP/latency ratio for the occurrence of atrial arrhythmias, as there were overlapping values in arrhythmic and non-arrhythmic hearts. Nevertheless, a pro-arrhythmic state was associated with a shorter ERP/latency ratio and vice versa in both the atria and ventricles. The length of the mouse atria is about 3 mm [52], which should only accommodate a re-entrant circuit with a circumference of around 9 mm. This is consistent with our estimated wavelength of 6 mm (using a distance between the stimulating and recording electrodes of 3 mm), implying that circus-type is indeed possible. In mice with over-expression of TGF-β1, atrial fibrillation was associated with a shorter wavelength compared to wild-type (15 mm vs. 28 mm, respectively) [52]. However, there was accompanying selective atrial fibrosis, which would increase the heterogeneity of conduction and allow micro-re-entry that can occur in smaller areas [53]. Without data from optical mapping, it was not possible to determine the electrophysiological mechanisms underlying AT observed in this study. However, previous experiments conducted in mouse hearts in non-exercised vs exercised mice have provided much insight, demonstrating possible roles of rotor formation [51].”

These findings confirmed the original hypothesis that atrial electrophysiological and arrhythmic properties differed from their ventricular counterparts both before and after application of heptanol. These differences are summarized in Table 2, explicable by the ERP/latency ratio.

**Table 2:** Comparisons of atrial and ventricular arrhythmic and electrophysiological properties before and after heptanol treatment.

	Heptanol—ventricles	Heptanol—atria	
Spontaneous arrhythmias	Observed during regular pacing with heptanol	Controls—none; heptanol—none	Controls—none; heptanol—none
Inducible arrhythmias	Controls—none;	Controls—non-arrhythmic at baseline;	Controls—atrial tachy-arrhythmias at baseline;
	Heptanol—Ventricular tachy-arrhythmias	Heptanol did not alter inducible arrhythmogenesis	Heptanol was anti-arrhythmic
Directly measured parameters			
Activation latency	↑↑	↑ (Note: non-arrhythmic group had significantly slower CV than arrhythmic hearts at baseline)	↑
ERP	↑	↑ (Note, non-arrhythmic group had significantly greater ERPs than arrhythmic hearts at baseline)	↑↑
APD_90_	Unchanged	Unchanged	Unchanged
Derived parameters			
ERP/latency	↓	Unchanged	↑ (Notes: [1] ERP/latency in arrhythmic group significantly less than in non-arrhythmic group at baseline; [2] heptanol increased ERP/latency in arrhythmic group to a value similar to the initially non-arrhythmic hearts)
ERP/APD_90_	↑	↑ (Note: ERP/APD_90_ starts off higher than in the arrhythmic group. It was not altered by heptanol)	↑↑ (Note: ERP/APD_90_ starts off lower in this group. Heptanol increases significantly it to a value approximating that of the untreated hearts in the non-arrhythmic group)
Reversibility	Reversible	Reversible	Reversible
Concentration dependence	2 mM: pro-arrhythmic and alters EP parameters;	2 mM: no change in arrhythmogenicity and alters EP parameters;	2 mM: anti-arrhythmic and alters EP parameters;
	0.1 to 1 mM heptanol not pro-arrhythmic, alters CV and ERP but not APD, ERP/latency or ERP/APD_90_	0.1 to 1 mM had no effect on any of the parameters	0.1 to 1 mM had no effect on any of the parameters

## Supporting Information

S1 Dataset Excel file of dataset on the arrhythmic and non-arrhythmic hearts: activation latency, CV, AERP, APD, AERP/latency and AERP/APD ratios. (XLSX)

## References

[1] Boyett MR. 'And the beat goes on.' The cardiac conduction system: the wiring system of the heart. Exp Physiol. 2009;94(10):1035–49. 10.1113/expphysiol.2009.046920 19592411

[2] Jansen JA, van Veen TA, de Bakker JM, van Rijen HV. Cardiac connexins and impulse propagation. J Mol Cell Cardiol 2010;48(1):76–82. 10.1016/j.yjmcc.2009.08.018 19729017

[3] Shaw RM, Rudy Y. Ionic mechanisms of propagation in cardiac tissue. Roles of the sodium and L-type calcium currents during reduced excitability and decreased gap junction coupling. Circ Res. 1997;81(5):727–41. 9351447 10.1161/01.res.81.5.727

[4] Sepp R, Severs NJ, Gourdie RG. Altered patterns of cardiac intercellular junction distribution in hypertrophic cardiomyopathy. Heart. 1996;76(5):412–7. Epub 1996/11/01. 8944586 10.1136/hrt.76.5.412 PMC484572

[5] Antzelevitch C. Ion channels and ventricular arrhythmias: cellular and ionic mechanisms underlying the Brugada syndrome. Curr Opin Cardiol. 1999;14(3):274–9. Epub 1999/06/08. . 10358800 10.1097/00001573-199905000-00013

[6] Sabir IN, Killeen MJ, Grace AA, Huang CL. Ventricular arrhythmogenesis: Insights from murine models. Prog Biophys Mol Biol. 2008;98:208–18. 10.1016/j.pbiomolbio.2008.10.011 19041335

[7] Gellens ME, George ALJ, Chen LQ, Chahine M, Horn R, Barchi RL, et al Primary structure and functional expression of the human cardiac tetrodotoxin-insensitive voltage-dependent sodium channel. Proc Natl Acad Sci U S A. 1992;89(2):554–8. 1309946 10.1073/pnas.89.2.554 PMC48277

[8] Berul CI. Electrophysiological phenotyping in genetically engineered mice. Physiol Genomics. 2003;13(3):207–16. 12746465 10.1152/physiolgenomics.00183.2002

[9] Killeen MJ, Thomas G, Sabir IN, Grace AA, Huang CL. Mouse models of ventricular arrhythmias. Acta Physiol (Oxf). 2008;192(4):455–69. 18045245 10.1111/j.1748-1716.2007.01822.x

[10] Tse G, Hothi SS, Grace AA, Huang CL. Ventricular arrhythmogenesis following slowed conduction in heptanol-treated, Langendorff-perfused mouse hearts. J Physiol Sci. 2012;62(2):79–92. 10.1007/s12576-011-0187-2 22219003 PMC10717265

[11] Balasubramaniam R, Grace AA, Saumarez RC, Vandenberg JI, Huang CL. Electrogram prolongation and nifedipine-suppressible ventricular arrhythmias in mice following targeted disruption of KCNE1. J Physiol. 2003;552(pt 2):535–46. 14561835 10.1113/jphysiol.2003.048249 PMC2343378

[12] Head CE, Balasubramaniam R, Thomas G, Goddard CA, Lei M, Colledge WH, et al Paced electrogram fractionation analysis of arrhythmogenic tendency in DeltaKPQ Scn5a mice. J Cardiovasc Electrophysiol. 2005;16(12):1329–40. 16403066 10.1111/j.1540-8167.2005.00200.x

[13] Dautova Y, Zhang Y, Grace AA, Huang CL. Atrial arrhythmogenic properties in wild-type and Scn5a+/- murine hearts. Exp Physiol. 2010;95(10):994–1007. 10.1113/expphysiol.2010.053868 20621962

[14] Dautova Y, Zhang Y, Sabir I, Grace AA, Huang CL. Atrial arrhythmogenesis in wild-type and Scn5a+/delta murine hearts modelling LQT3 syndrome. Pflugers Arch 2009;458(3):443–57. 10.1007/s00424-008-0633-z 19184093 PMC2691533

[15] Knollmann BC, Katchman AN, Franz MR. Monophasic action potential recordings from intact mouse heart: validation, regional heterogeneity, and relation to refractoriness. J Cardiovasc Electrophysiol 2001;12:1286–94. 11761418 10.1046/j.1540-8167.2001.01286.x

[16] Gussak I, Chaitman BR, Kopecky SL, Nerbonne JM. Rapid ventricular repolarization in rodents: electrocardiographic manifestations, molecular mechanisms, and clinical insights. J Electrocardiol 2000;33:159–70. 10819409 10.1016/s0022-0736(00)80072-2

[17] Fabritz L, Kirchhof P, Franz MR, Eckardt L, Mönnig G, Milberg P, et al Prolonged action potential durations, increased dispersion of repolarization, and polymorphic ventricular tachycardia in a mouse model of proarrhythmia. Basic Res Cardiol. 2003;98(1):25–32. 12494266 10.1007/s00395-003-0386-y

[18] Sun D, Samuelson LC, Yang T, Huang Y, Paliege A, Saunders T, et al Mediation of tubuloglomerular feedback by adenosine: evidence from mice lacking adenosine 1 receptors. Proc Natl Acad Sci U S A 2001;98(17):9983–8. 11504952 10.1073/pnas.171317998 PMC55564

[19] Tse G, Yeo JM. Conduction abnormalities and ventricular arrhythmogenesis: The roles of sodium channels and gap junctions. IJC Heart & Vasculature. 2015;9:75–82. 10.1016/j.ijcha.2015.10.003 26839915 PMC4695916

[20] Sheikh SM, Skepper JN, Chawla S, Vandenberg JI, Elneil S, Huang CL. Normal conduction of surface action potentials in detubulated amphibian skeletal muscle fibres. J Physiol. 2001;535(Pt 2):579–90. Epub 2001/09/05. doi: PHY_12114 [pii]. 11533146 10.1111/j.1469-7793.2001.t01-1-00579.x PMC2278804

[21] Antzelevitch C, Burashnikov A. Overview of Basic Mechanisms of Cardiac Arrhythmia. Cardiac Electrophysiology Clinics. 2011;3(1):23–45. 21892379 10.1016/j.ccep.2010.10.012 PMC3164530

[22] Tse G. Mechanisms of Cardiac Arrhythmias. Journal of Arrhythmia. 2015 10.1016/j.joa.2015.11.003 PMC4823581 27092186

[23] Smeets JL, Allessie MA, Lammers WJ, Bonke FI, Hollen J. The wavelength of the cardiac impulse and reentrant arrhythmias in isolated rabbit atrium. The role of heart rate, autonomic transmitters, temperature, and potassium. Circ Res. 1986;58(1):96–108. 3943157 10.1161/01.res.58.1.96

[24] Wiener N, Rosenblueth A. The mathematical formulation of the problem of conduction of impulses in a network of connected excitable elements, specifically in cardiac muscle. Arch Inst Cardiol Mex. 1946;16(3):205–65. 20245817

[25] Thomas GP, Howlett SE, Ferrier GR. Saralasin suppresses arrhythmias in an isolated guinea pig ventricular free wall model of simulated ischemia and reperfusion. J Pharmacol Exp Ther. 1995;274(3):1379–86. Epub 1995/09/01. . 7562511

[26] Sabir IN, Fraser JA, Killeen MJ, Grace AA, Huang CL. The contribution of refractoriness to arrhythmic substrate in hypokalaemic Langendorff-perfused murine hearts. Pflugers Arch. 2007;454:209–22. 17295037 10.1007/s00424-007-0217-3 PMC1839769

[27] Antzelevitch C. In vivo human demonstration of phase 2 reentry. Heart Rhythm. 2005;2(8):804–6. Epub 2005/07/30. doi: S1547-5271(05)01663-2 [pii] 10.1016/j.hrthm.2005.05.013 16051113 PMC1474078

[28] Brugada P, Wellens HJ. Early afterdepolarizations: role in conduction block, "prolonged repolarization-dependent reexcitation," and tachyarrhythmias in the human heart. Pacing Clin Electrophysiol. 1985;8(6):889–96. 2415942 10.1111/j.1540-8159.1985.tb05908.x

[29] Li G, Whittaker P, Yao M, Kloner RA, Przyklenk K. The gap junction uncoupler heptanol abrogates infarct size reduction with preconditioning in mouse hearts. Cardiovasc Pathol. 2002;11(3):158–65. Epub 2002/05/29. doi: S1054880702001023 [pii]. . 12031768 10.1016/s1054-8807(02)00102-3

[30] Chen BP, Mao HJ, Fan FY, Bruce IC, Xia Q. Delayed uncoupling contributes to the protective effect of heptanol against ischaemia in the rat isolated heart. Clin Exp Pharmacol Physiol. 2005;32(8):655–62. 16120193 10.1111/j.0305-1870.2005.04246.x

[31] Keevil VL, Huang CL, Chau PL, Sayeed RA, Vandenberg JI. The effect of heptanol on the electrical and contractile function of the isolated, perfused rabbit heart. Pflügers Arch. 2000;440:275–82. 10898528 10.1007/s004240000264

[32] Boersma L, Brugada J, Abdollah H, Kirchhof C, Allessie M. Effects of heptanol, class Ic, and class III drugs on reentrant ventricular tachycardia. Importance of the excitable gap for the inducibility of double-wave reentry. Circulation. 1994;90(2):1012–22. Epub 1994/08/01. . 8044914 10.1161/01.cir.90.2.1012

[33] Ohara T, Qu Z, Lee MH, Ohara K, Omichi C, Mandel WJ, et al Increased vulnerability to inducible atrial fibrillation caused by partial cellular uncoupling with heptanol. Am J Physiol Heart Circ Physiol. 2002;283(3):H1116–22. 12181142 10.1152/ajpheart.00927.2001

[34] Bastiaanse EM, Jongsma HJ, van der Laarse A, Takens-Kwak BR. Heptanol-induced decrease in cardiac gap junctional conductance is mediated by a decrease in the fluidity of membranous cholesterol-rich domains. J Membr Biol 1993;136:135–45. 7508980 10.1007/BF02505758

[35] Nelson WL, Makielski JC. Block of sodium current by heptanol in voltage-clamped canine cardiac Purkinje cells. Circ Res. 1991;68:977–83. 1849060 10.1161/01.res.68.4.977

[36] Tse G, Tse V, Yeo JM. Ventricular anti-arrhythmic effects of heptanol in hypokalaemic, Langendorff-perfused mouse hearts. Biomedical Reports. 2015 10.3892/br.2016.577 PMC4774402 26998268

[37] Koller BS, Karasik PE, Solomon AJ, Franz MR. Relation between repolarization and refractoriness during programmed electrical stimulation in the human right ventricle. Implications for ventricular tachycardia induction. Circulation. 1995;91(9):2378–84. 7729024 10.1161/01.cir.91.9.2378

[38] Rozanski GJ, Jalife J, Moe GK. Determinants of postrepolarization refractoriness in depressed mammalian ventricular muscle. Circ Res. 1984;55(4):486–96. 6478553 10.1161/01.res.55.4.486

[39] Temple IP, Inada S, Dobrzynski H, Boyett MR. Connexins and the atrioventricular node. Heart Rhythm. 2013;10(2):297–304. 10.1016/j.hrthm.2012.10.020 23085482 PMC3572393

[40] Remme CA, Verkerk AO, Hoogaars WM, Aanhaanen WT, Scicluna BP, Annink C, et al The cardiac sodium channel displays differential distribution in the conduction system and transmural heterogeneity in the murine ventricular myocardium. Basic Res Cardiol. 2009;104(5):511–22. 10.1007/s00395-009-0012-8 19255801 PMC2722719

[41] Li GR, Lau CP, Shrier A. Heterogeneity of sodium current in atrial vs epicardial ventricular myocytes of adult guinea pig hearts. J Mol Cell Cardiol. 2002;34(9):1185–94. . 12392892 10.1006/jmcc.2002.2053

[42] Schroeter A, Walzik S, Blechschmidt S, Haufe V, Benndorf K, Zimmer T. Structure and function of splice variants of the cardiac voltage-gated sodium channel Na(v)1.5. J Mol Cell Cardiol. 2010;49(1):16–24. 10.1016/j.yjmcc.2010.04.004 . 20398673

[43] Fahmi AI, Patel M, Stevens EB, Fowden AL, John JE III, Lee K, et al The sodium channel beta-subunit SCN3b modulates the kinetics of SCN5a and is expressed heterogeneously in sheep heart. J Physiol. 2001;537(Pt 3):693–700. 11744748 10.1111/j.1469-7793.2001.00693.x PMC2278985

[44] Abriel H. Cardiac sodium channel Na(v)1.5 and interacting proteins: Physiology and pathophysiology. J Mol Cell Cardiol. 2010;48(1):2–11. 10.1016/j.yjmcc.2009.08.025 . 19744495

[45] Beyer EC, Paul DL, Goodenough DA. Connexin43: a protein from rat heart homologous to a gap junction protein from liver. J Cell Biol. 1987;105(6 Pt 1):2621–9. Epub 1987/12/01. 2826492 10.1083/jcb.105.6.2621 PMC2114703

[46] Davis LM, Kanter HL, Beyer EC, Saffitz JE. Distinct gap junction protein phenotypes in cardiac tissues with disparate conduction properties. J Am Coll Cardiol. 1994;24(4):1124–32. Epub 1994/10/01. doi: 0735-1097(94)90879-6 [pii]. . 7930207 10.1016/0735-1097(94)90879-6

[47] Gros D, Jarry-Guichard T, Ten Velde I, de Maziere A, van Kempen MJ, Davoust J, et al Restricted distribution of connexin40, a gap junctional protein, in mammalian heart. Circ Res. 1994;74(5):839–51. . 8156631 10.1161/01.res.74.5.839

[48] Lin X, Gemel J, Glass A, Zemlin CW, Beyer EC, Veenstra RD. Connexin40 and connexin43 determine gating properties of atrial gap junction channels. J Mol Cell Cardiol. 2010;48(1):238–45. 10.1016/j.yjmcc.2009.05.014 19486903 PMC2813328

[49] Alcolea S, Jarry-Guichard T, de Bakker J, Gonzalez D, Lamers W, Coppen S, et al Replacement of connexin40 by connexin45 in the mouse: impact on cardiac electrical conduction. Circ Res. 2004;94(1):100–9. 10.1161/01.RES.0000108261.67979.2A . 14630724

[50] Arora R, Das MK, Zipes DP, Wu J. Optical mapping of cardiac arrhythmias. Indian Pacing Electrophysiol J. 2003;3(4):187–96. 16943918 PMC1502051

[51] Aschar-Sobbi R, Izaddoustdar F, Korogyi AS, Wang Q, Farman GP, Yang F, et al Increased atrial arrhythmia susceptibility induced by intense endurance exercise in mice requires TNFalpha. Nat Commun. 2015;6:6018 10.1038/ncomms7018 . 25598495 PMC4661059

[52] Verheule S, Sato T, Everett T, Engle SK, Otten D, Rubart-von der Lohe M, et al Increased vulnerability to atrial fibrillation in transgenic mice with selective atrial fibrosis caused by overexpression of TGF-beta1. Circ Res. 2004;94(11):1458–65. 15117823 10.1161/01.RES.0000129579.59664.9d PMC2129102

[53] Spach MS, Josephson ME. Initiating reentry: the role of nonuniform anisotropy in small circuits. J Cardiovasc Electrophysiol. 1994;5(2):182–209. Epub 1994/02/01. . 8186887 10.1111/j.1540-8167.1994.tb01157.x

